# Psychosis in a Military Veteran in the Context of Hallucinogen Use: A Case Report

**DOI:** 10.7759/cureus.79308

**Published:** 2025-02-19

**Authors:** Paria Parhami, Sydney E Moriarty, Jay Parikh, Linda Galicki

**Affiliations:** 1 Psychiatry, Edward Via College of Osteopathic Medicine, Blacksburg, USA; 2 Psychiatry, LewisGale Medical Center, Salem, USA; 3 Psychiatry, Salem Veteran Affairs Medical Center, Salem, USA

**Keywords:** delusional parasitosis- clinical, dimethyltryptamine, dmt-induced delusional parasitosis, dmt-induced psychosis, drug-induced psychosis

## Abstract

N, N-dimethyltryptamine (DMT) is a potent hallucinogenic compound with increasing recreational use despite limited safety data. While DMT use has been linked to psychosis, delusional parasitosis as a specific manifestation remains unreported. We present a novel case of a 60-year-old female veteran with chronic post-traumatic stress disorder (PTSD), a history of major depressive disorder, past marijuana use, and no history of prior psychiatric hospitalizations, who developed persistent delusions of parasitic infestation following prolonged recreational intravenous (IV) DMT. She was referred to acute inpatient psychiatry by the residential PTSD program in the same hospital system, where she expressed upon intake there the strong conviction that worms were coming out of her skin. Upon admission to inpatient psychiatry, the patient presented with severe anxiety about minor appearing skin excoriations. These were likely a result of and consequent precipitator of her delusion that a parasite-infected her eight months ago during a plumbing leak in her home. A review of her records revealed endorsement of these symptoms to multiple providers over the past eight months. She also reported using IV DMT at home 1-2 times per month for the past 18 months. As the timing of hallucinogen use and the development of psychotic symptoms were not fully consistent, there was a concern for a primary delusional disorder that was likely exacerbated by her substance use. Her delusions persisted despite initial haloperidol treatment initiated during hospitalization but improved after 15 days of hospitalization, following substance clearance and psychoeducation. This case suggests a novel potential link between prolonged DMT use and delusional parasitosis, as no published studies have specifically explored this association, highlighting the need for further research, particularly with repeated and IV administration.

## Introduction

N, N-dimethyltryptamine (DMT) is a naturally occurring, potent hallucinogenic compound that acts primarily as a partial agonist on 5-hydroxytryptamine 1A, 2A, and 2C receptors to induce rapid changes in perception [1]. The hallucinogen is commonly extracted from ayahuasca, an herbal product derived from the Banisteriopsis caapi vine (beta-carboline harmala alkaloids) and leaves of the Psychotria viridis bush (which contains DMT). Ayahuasca has been traditionally used for over 3,000 years by indigenous shamans to facilitate religious and spiritual healing. In recent years, however, its use has expanded to include recreational hallucinogen consumption in Europe and North America [2].

DMT use typically induces intense visual and auditory hallucinations, altered perceptions of time and space, and profound changes in consciousness. Users often report experiences of entering alternate realities or encountering otherworldly beings. DMT can be administered via intravenous (IV), intramuscular (IM), inhalation (smoking or vaporization), or oral ingestion. When taken orally, monoamine oxidase inhibitors (MAOIs), like harmala alkaloids from ayahuasca, are necessary to prevent DMT's breakdown in the digestive system. IV administration, however, carries higher risks due to the rapid onset of effects and peak DMT blood levels within minutes, leading to high bioavailability [3]. DMT has a short half-life, with plasma levels rapidly decreasing within minutes of intravenous administration, typically ranging from 5 to 15 minutes [3]. This increases the potential for acute toxicity and the risk of serotonin syndrome, especially when combined with other serotonergic substances. Limited evidence exists on the safety profile of DMT, particularly regarding IV administration and the risks associated with repeated or cumulative exposure, despite its increasing popularity for recreational use. One study estimated the lifetime prevalence of hallucinogen use at 9%, with LSD at 39% and psilocybin at 43%, while DMT attracts the highest proportion of new users, reflecting its increasing interest [4]. A recent systematic review identified three documented cases of psychosis linked to recreational DMT use, all involving concurrent substance use and a family history of psychosis [5].

We present a unique case of a female patient with post-traumatic stress disorder (PTSD) who experienced an undocumented psychotic symptom-delusional parasitosis following prolonged DMT use. Notably, she had a history of cannabis use but no use for the past 14 months. This case highlights a potentially unique association between DMT use and the development of delusional parasitosis. Unlike other cases of DMT-related psychosis, which typically involve sensory hallucinations or disorganized behavior, the patient's delusions were specifically focused on parasitic infestation, presenting a novel manifestation of substance-induced psychosis. Overall, a recent systematic review did not find any documented associations between hallucinogens and delusional parasitosis. In fact, the only recreational drugs linked to delusional parasitosis in the review were cocaine, amphetamines, and amphetamine-like substances such as pemoline, atomoxetine, and methylphenidate [6]. This suggests that DMT use may present a distinct risk for the development of delusional parasitosis, an association that warrants further investigation.

## Case presentation

The patient was a 60-year-old female military veteran with chronic PTSD and a history of depression, with no prior psychiatric hospitalizations, who was referred to inpatient psychiatry in November 2023 after being at the residential PTSD program for one day, where she expressed strong convictions that worms were under her skin, prompting psychiatric evaluation. She had been admitted to the PTSD program for treatment of long-standing interpersonal issues. This was the patient's first time attending a residential program for treating PTSD, and she had been on chronic medical therapy for PTSD, taking venlafaxine 150 mg. Upon admission to inpatient psychiatry, the patient presented with severe anxiety about minor-appearing skin excoriations on her left-hand fingers, dorsal forearms, and anterior thighs. She believed a parasite had infected her eight months ago during a plumbing leak in her home. Records showed that she had reported these symptoms to multiple providers over the past eight months. During this time, she was prescribed two different atypical antipsychotics, quetiapine 50 mg and aripiprazole 1 mg, to address the delusion, but she stopped taking them shortly after starting each one within about a month due to experiencing enuresis as a side effect. She also reported using 25 milliliters of IV DMT at home once a week for the past 18 months. The patient admitted to using DMT recreationally alongside her partner, who was using it to cope with his terminal cancer diagnosis. She denied any other substance use, except for intermittent marijuana use in the past, which had not occurred for the past 13 months.

Organic causes of psychosis were considered and ruled out through an initial medical workup. Dermatology was consulted for additional diagnostic clarity and to assess for any underlying dermatological condition. No burrows were identified, and the discrete nature of lesions on readily accessible sites on her anterior thighs and distal forearms was suggestive of external digitization. The patient was diagnosed with eczematous dermatitis without evidence of parasite infestation. Given the absence of dermatological findings consistent with a parasitic infestation, the dermatological diagnosis was ruled out, and her skin symptoms were instead attributed to a psychiatric diagnosis of delusional disorder. She was prescribed haloperidol 2 mg twice a day to address distressing delusions, along with topical treatments recommended by dermatology, including mupirocin, tacrolimus, and clobetasol. A first-generation antipsychotic like haloperidol was chosen to see if she would respond better after failing two previous trials of second-generation antipsychotics. However, haloperidol was discontinued after five days when the patient reported significant drowsiness as a side effect. The patient declined further antipsychotic treatment, and her delusions did not improve. Over the course of her hospitalization, with repeated psychoeducation and review of dermatologic findings, which included a second dermatology consult and evaluation, she became more accepting of her diagnosis. This was also likely related to the clearance of DMT based on its fast metabolism and clearance [7,8]. She gradually expressed less distress from her delusions, although they had not fully resolved. She was discharged after 15 days of hospitalization.

## Discussion

This case illustrates the association between repeated DMT use and the development of psychotic symptoms, specifically delusional parasitosis. We demonstrate an improvement in symptoms with repeated psychoeducation and allowing sufficient time for substance clearance. While several other instances of psychosis related to DMT have been documented in the literature, none featured delusional parasitosis. In comparison to the cases described in the literature, the psychotic symptoms in this patient differ in key aspects. The patient in this case, like the subjects in the cited cases, experienced delusions and distressing beliefs. However, this patient's delusions were more specifically focused on parasitic infestation rather than the grandiose or spiritual delusions observed in those cases (e.g., believing the individual was a king or interacting with aliens). Additionally, while the patients in the literature had more generalized psychotic episodes with sensory hallucinations and disorganized behavior, this patient's delusions were more confined to a specific, persistent belief about skin infestations.

The patient, in this case, admitted to using marijuana intermittently, with the last use occurring 13 months prior to admission. This timeframe overlaps with her 18 months of regular DMT use, making it unclear whether marijuana use initiated or exacerbated the initial onset of psychotic symptoms. Like the findings in documented cases in the literature, where chronic marijuana use was identified as a potential trigger for psychosis, the patient's past marijuana use may have increased her vulnerability to developing psychotic symptoms. However, given the more prominent and sustained use of DMT in the patient's history, it is difficult to definitively determine the role of marijuana in the onset or worsening of her delusions. As seen in the cases documented in the literature, the interaction between marijuana and other substances like DMT may have compounded the psychotic episode, but the primary influence seems to stem from DMT use.

Additionally, the impact of IV administration of DMT on its psychoactive effects remains largely uncertain. One study revealed that levels of plasma DMT differed among individuals who received the same single dose of IV DMT, indicating that an individual's unique pharmacokinetics could affect their psychological reaction [9]. This is important because differences in metabolism can influence both the intensity and duration of DMT's effects, potentially leading to varied psychiatric outcomes. For example, slower clearance could result in prolonged effects, increasing the risk of persistent psychosis. Repeated IV DMT use may also lead to cumulative exposure, where DMT accumulates in the brain over time, particularly in individuals who use it frequently. This cumulative effect could exacerbate psychotic symptoms or contribute to the chronicity of delusions, raising concerns for long-term psychiatric impact. In rabbits, radiolabeled DMT persisted in the brain for as long as seven days following injection, suggesting that repeated administration could lead to a gradual accumulation of the substance in the brain, potentially contributing to the development of psychosis over time [9,10].

While the mechanism linking DMT to delusional parasitosis remains speculative, several hypotheses could explain this association. One possibility is serotonin receptor dysregulation. DMT, as a serotonin receptor agonist, may induce disturbances in serotonin signaling, which could contribute to altered perception, misidentifications, and delusions, such as those related to parasitic infestation. Another potential mechanism involves trauma-triggered fixation. The patient's history of chronic PTSD may have predisposed her to fixate on an external threat, such as parasitic infection, as a way of coping with her trauma.

One limitation in this case is the absence of metabolite testing to confirm DMT clearance, which would have provided a clearer understanding of its pharmacokinetics and role in the persistence of psychotic symptoms. Additionally, the short follow-up period limits our ability to assess the long-term effects of repeated DMT use on the patient's psychiatric health. These limitations underscore the need for further research to validate the associations observed in this case. Future studies could explore the mechanisms of DMT-induced psychosis, including its interaction with serotonin receptors. Studies examining individual differences in DMT metabolism and clearance and controlled metabolite testing would help clarify the variability of psychiatric outcomes.

## Conclusions

This case highlights the potential for repeated intravenous DMT use to contribute to the development of psychotic symptoms, including delusional parasitosis. The improvement in symptoms observed after cessation of DMT use emphasizes the importance of early recognition and intervention. Given the increasing recreational use of DMT, further research is essential to better understand its safety profile, especially in the context of its method of administration and long-term psychiatric effects.
